# Case Report: Electroencephalography in a neonate with isolated sulfite oxidase deficiency
 – a case report and literature review

**DOI:** 10.12688/hrbopenres.13442.1

**Published:** 2021-11-23

**Authors:** Andreea M Pavel, Carol M Stephens, Sean R Mathieson, Brian H Walsh, Brian McNamara, Niamh McSweeney, Geraldine B Boylan

**Affiliations:** 1 INFANT Research Centre, University College Cork, Cork, Ireland; 2 Department of Paediatrics and Child Health, University College Cork, Cork, Ireland; 3 Department of Neonatology, Cork University Maternity Hospital, Cork, Ireland; 4 Department of Neurophysiology, Cork University Hospital, Cork, Ireland; 5 Department of Paediatric Neurology, Cork University Hospital, Cork, Ireland

**Keywords:** neonatal seizures; encephalopathy; electroencephalography; sulfite oxidase deficiency; refractory seizures; brain MRI; case report

## Abstract

Isolated sulfite oxidase deficiency (ISOD) is a rare autosomal recessive neuro-metabolic disorder caused by a mutation in the sulfite oxidase (SUOX) gene situated on chromosome 12. Due to the deficiency of this mitochondrial enzyme (sulfite oxidase), the oxidative degradation of toxic sulfites is disrupted. The most common form of this disease has an early onset (classical ISOD) in the neonatal period, with hypotonia, poor feeding and intractable seizures, mimicking hypoxic-ischaemic encephalopathy. The evolution is rapidly progressive to severe developmental delay, microcephaly and early death. Unfortunately, there is no effective treatment and the prognosis is very poor.

In this article, we described the evolution of early continuous electroencephalography (EEG) in a case of ISOD with neonatal onset, as severely encephalopathic background, with refractory seizures and distinct delta-beta complexes. The presence of the delta-beta complexes might be a diagnostic marker in ISOD. We also performed a literature review of published cases of neonatal ISOD that included EEG monitoring.

## Introduction

Early onset isolated sulfite oxidase deficiency (ISOD) is a rare neuro-metabolic disease affecting infants in newborn period, with a very poor prognosis. Due to the early onset with encephalopathy and seizures, it is very important to differentiate ISOD from other causes of encephalopathy in newborns, mainly hypoxic-ischaemic encephalopathy for which early intervention with therapeutic hypothermia is crucial.

There are just a few cases with ISOD presented in the literature and even fewer with description of the electroencephalographic pattern. We present the case of a newborn with ISOD, and we describe in detail the evolution of the electroencephalographic background and electrographic seizure activity, suggesting that the presence of specific delta-beta complexes might be a diagnostic marker of this disease.

## Case presentation

We present the case of a term female infant born (Apgars 9 and 9 at 1 and 5 minutes, respectively) by elective caesarean section for expected macrosomia, to non-consanguineous Caucasian parents. Medical history of note included maternal gestational diabetes treated with metformin, otherwise antenatal and family histories were unremarkable. The infant was admitted to the Neonatal Unit at one hour of age with signs of respiratory distress requiring non-invasive respiratory support. In the first day of life, she was noted to have several episodes of crying with desaturation, associated with abnormal posturing (hypertonic extension of the body, trunk and limbs, occasional jerking of the limbs and a biting suck). Neurologic exam at the time revealed central hypotonia with peripheral hypertonia. Continuous electroencephalographic monitoring (cEEG) was commenced at approximatively 24 hours of age due to persisting episodes of abnormal movements, some of which were associated with desaturation.

**Figure 1.**
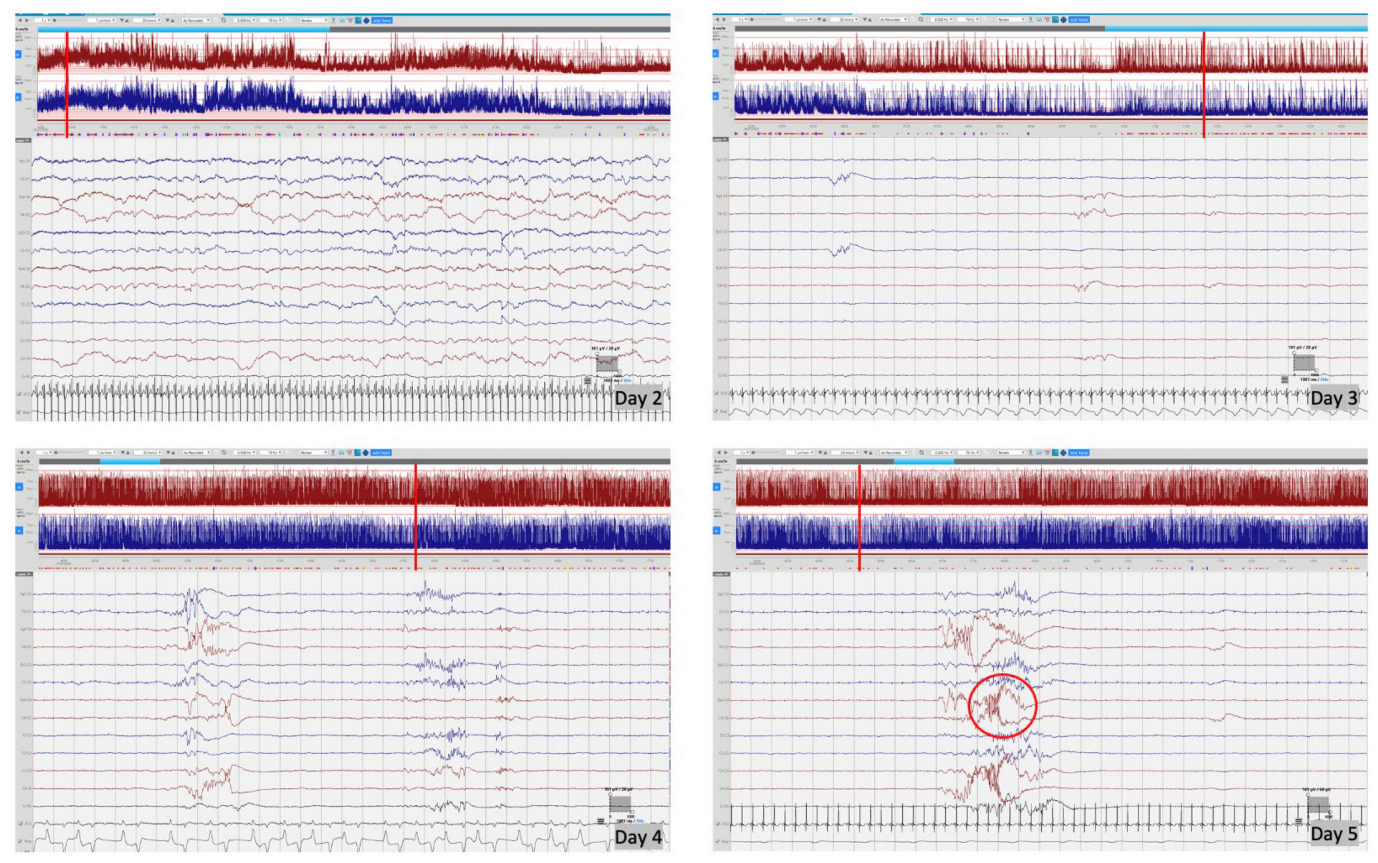
EEG background evolution in the first week of life. Day 2 of life – encephalopathic background with continuous delta activity with moderate theta activity, but paucity of fast frequencies, no sleep cycling; Day 3 of life – highly suppressed background activity post escalation of anti-seizure treatment, showing only low amplitude asynchronous transients of 1–2 sec against a highly suppressed background; Day 4 of life – some return of background activity with longer bursts of approximately 5 seconds duration but background remaining highly discontinuous with inter-burst intervals >20 seconds; Day 5 of life – remaining highly discontinuous with increased frequency of mechanical brushes/delta crown (circled image). Red vertical line indicates the position of EEG selection on the aEEG trace.

**Figure 2.**
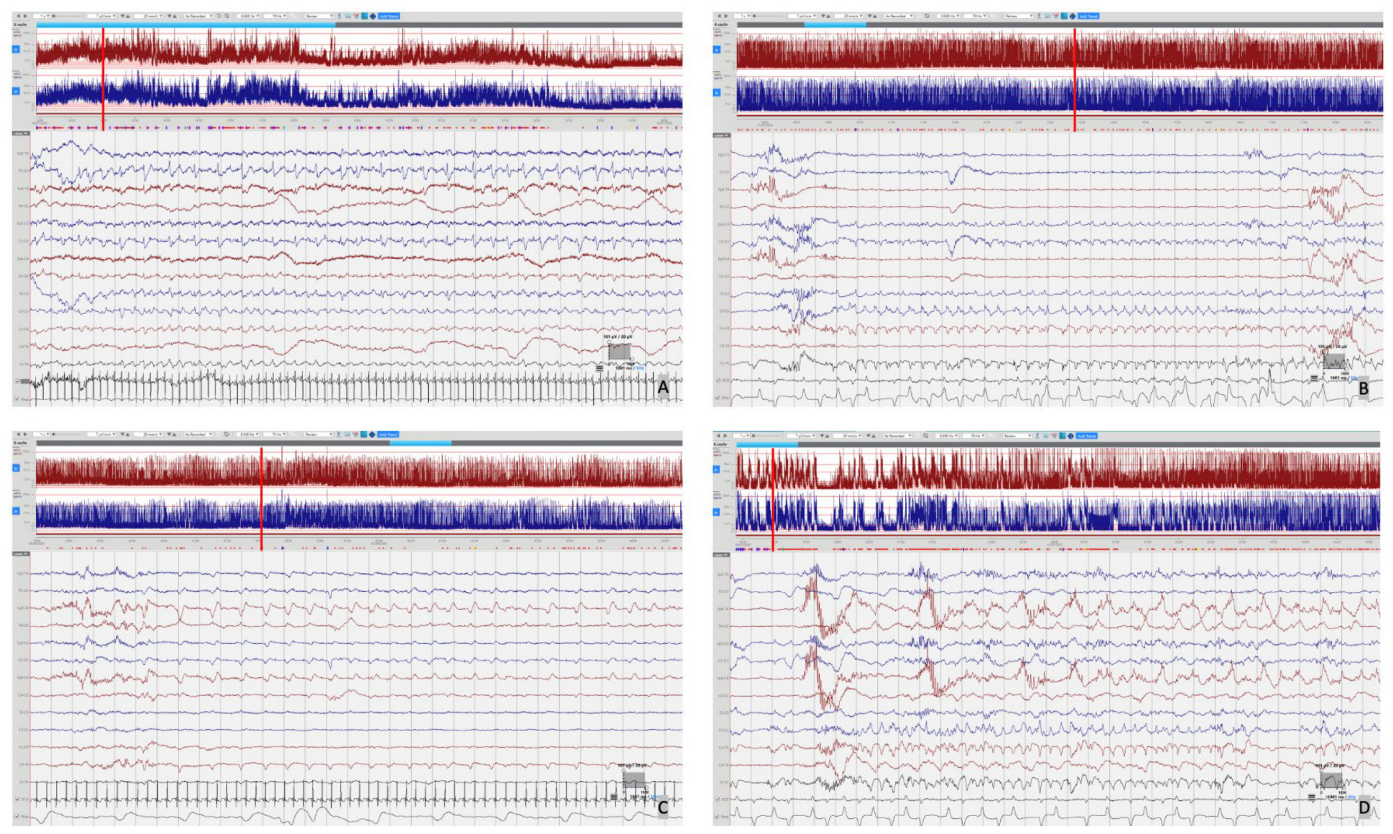
Seizures. Focal seizures were seen over several regions with variable seizure morphology including: **A**) Left sided seizure with sharp wave/slow wave morphology. **B**) 2 Hz delta seizure over the midline. **C**)~1 Hz delta seizure over the right anterior quadrant. **D**) Concurrent multifocal seizures with ~2Hz delta seizure over the central midline and ~1 Hz delta seizure over the right anterior. Red vertical line indicates the position of EEG selection on the aEEG trace.

**Figure 3.**
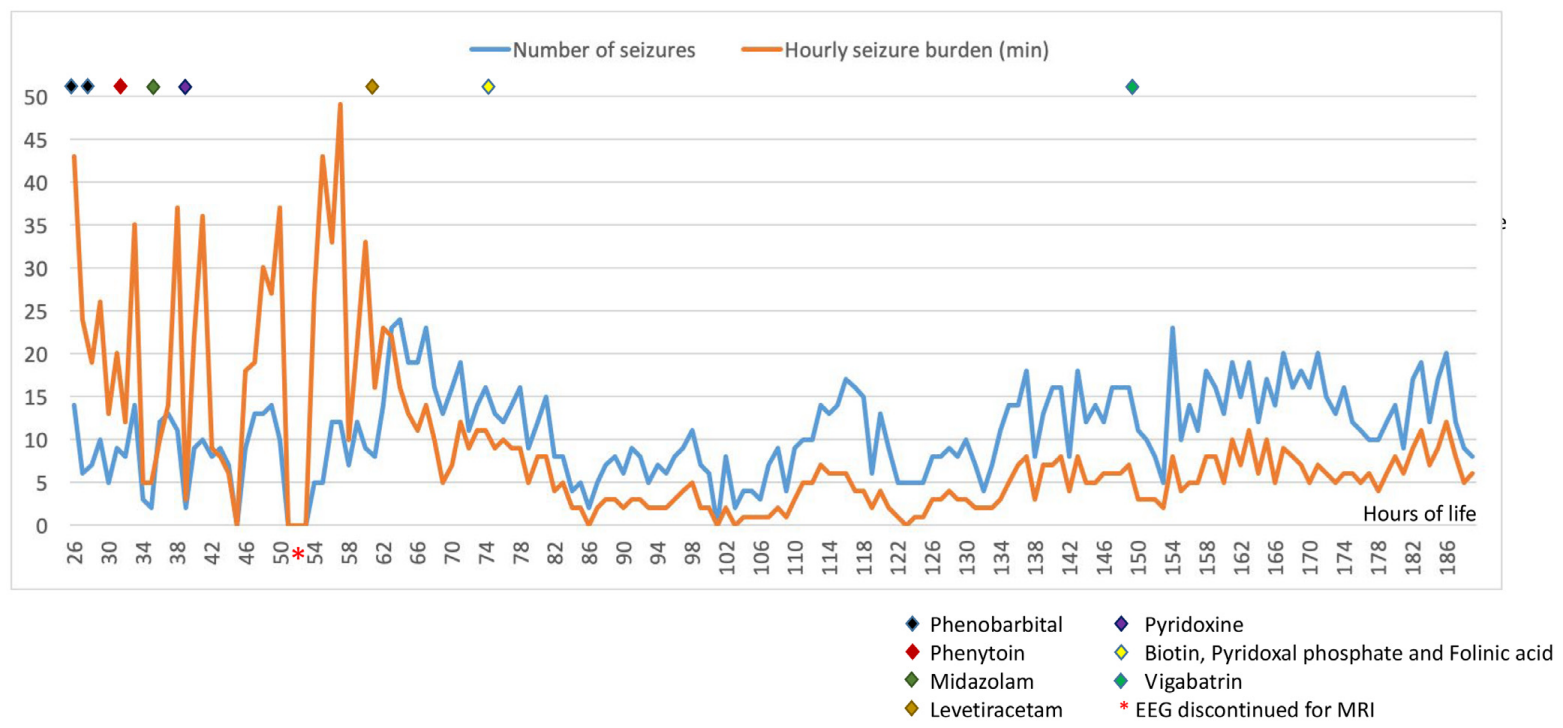
Evolution of hourly seizure burden and antiseizure management (loading/start doses) during first week of life.

**Figure 4.**
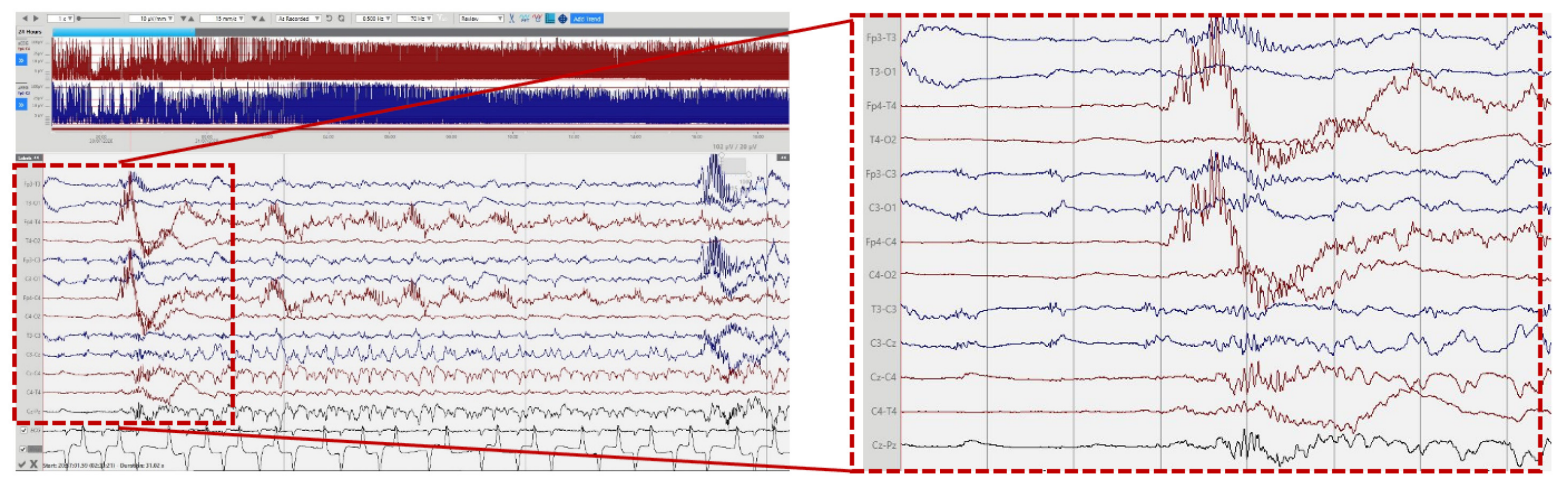
Delta-beta complexes. High voltage delta activity with overlying rhythmic fast activity at 10–25Hz, separated by periods of suppression of 10–30 seconds.

cEEG monitoring was performed using Lifelines iEEG (Lifelines Neuro, UK) with disposable electrodes positioned at Fp3, Fp4, C3, C4, Cz, Pz, T3, T4, O1, O2 according to the International 10:20 EEG electrode placement system adapted for neonates, with synchronous respiratory and heart rate monitoring. The initial EEG background pattern from 24 hours of age showed an encephalopathic pattern with persistent irregular delta activity and intermittent theta activity but with a paucity of faster frequencies and no sleep cycling. Frequent multifocal seizures were also seen. The evolution of the EEG background activity ( Figure 1) deteriorated over the first week of life, becoming more discontinuous and suppressed, which may be explained by the evolution of the disease but likely also due to the rapid escalation of anti-seizure treatment with partial response. EEG seizures consisted of either rhythmic delta patterns at 1–2Hz, or sharp wave/slow wave complexes with variable onset over the left hemisphere, right anterior quadrant or central midline, some with secondary generalisation and some occurring concurrently at times ( Figure 2). Seizure duration varied in the first few days between 30 seconds and 10 minutes, then mostly short (< 1min) thereafter. All electrographic seizures over the first week of life were annotated and the evolution of the hourly seizure burden and initial anti-seizure therapy is shown in Figure 3. A high seizure burden and status epilepticus (defined as at least 30 minutes of seizures within one hour) were noted up to 72 hours of life. Initially, most seizures were tonic (tonic extension of the body and upper limbs), some seizures were also associated with an altered breathing pattern, with desaturation, cycling of the upper and lower limbs or jittery limb movements. In day two of life, loading doses of Phenobarbitone (2x20mg/kg/day) and Phenytoin (20mg/kg/day) were given in quick succession from onset of EEG monitoring, during which time the background EEG became increasingly discontinuous with refractory multifocal seizures. Electroclinical seizures persisted, therefore commenced treatment with Midazolam infusion (150 micrograms/kg loading, continued with infusion titrated between 1–3 micrograms/kg/minute) and then Levetiracetam (10mg/kg loading increased to 30mg/kg BD) with partial response ( Figure 3). The rapid escalation of antiseizure treatment (phenobarbital, phenytoin, midazolam) in day 2 of life resulted in decreased respiratory effort and the infant required to be intubated and mechanically ventilated for 15 days. In addition, seizures were now electrographic only (most likely due to anti-seizure medication, electroclinical dissociation). On day 3, the hourly seizure burden decreased despite an increase in the number of seizures per hour, compared with the previous days. Pyridoxine (loading 50mg, up to 50mg BD), Pyridoxal phosphate (10mg/kg TDS), Biotin (5mg up to 10mg TDS) and Folinic acid (2.5mg up to 5mg BD) supplemented the initial antiseizure treatment. The predominant patterns of the EEG recording were: (1) frequent and refractory multifocal seizures ( Figure 2); (3) bursts of synchronous, or more often asynchronous delta activity with overlying rhythmic fast activity at 10–25Hz, separated by periods of suppression of 10–30 seconds ( Figure 4). The bursts of delta with overlying fast activity resembled ‘mechanical brushes’ frequently seen in preterm infants ^1^. However, it is interesting to note that Flitton *et al.* ^2^ described a distinctive waveform of slow waves with superimposed 13–20Hz, termed ‘delta crowns’, in a cohort of 5 infants over the first 74 days of life with Molybdenum cofactor deficiency, a syndrome noted to have very similar presentation to ISOD.

**Figure 5.**
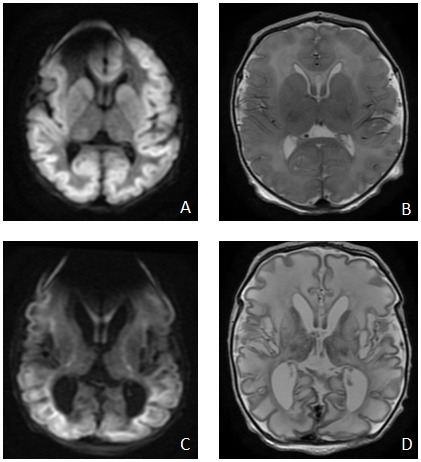
MRI Brain. on day of life 3 - **A** (DWI sequence) and **B** (axial T2): diffuse symmetrical abnormal restricted diffusion involving the cortical and subcortical regions of the cerebral hemispheres bilaterally, diffuse oedema of the white matter symmetrical abnormal diffusion restriction involving the caudate nuclei, lentiform nucleus, and dorsal thalami bilaterally; Repeat MRI Brain at 2 weeks of age – **C** (DWI sequence) and **D** (axial T2): interval development of cystic encephalomalacia with white matter and deep grey volume loss, persistent cortical/subcortical and deep grey abnormal diffusion restriction.

Brain magnetic resonance imaging (MRI) on day 3 ( Figure 5. A and B) showed diffuse symmetrical abnormal restricted diffusion involving the cortical and subcortical regions of the cerebral hemispheres bilaterally. Diffuse oedema of the white matter was also present with restricted diffusion extending along the corticospinal tracts to the level of the midbrain and also in the splenium in keeping with pre Wallerian degeneration. There was symmetrical abnormal diffusion restriction involving the caudate nuclei, lentiform nucleus, and dorsal thalami bilaterally, loss of the normal T2 hypointense signal in the posterolateral putamina bilaterally with loss of the normal T1 hyperintense signal in the posterior limb of the internal capsule and globus pallidus bilaterally. On magnetic resonance spectroscopy (MRS) a small lactate peak was noted. Repeat MRI at 2 weeks of age ( Figure 5. C and D) showed interval development of cystic encephalomalacia with white matter and deep gray volume loss; persistent cortical/subcortical and deep gray abnormal diffusion restriction and new cerebral venous sinus thrombosis in a posterior distribution.

A full metabolic and genetic panel was performed as a diagnostic workup in the first week of life (serum amino acids, urine organic acids, acylcarnitine, very low chain fatty acids, ammonia, homocysteine, sulfocysteine, pipecolic acid, alpha amino adipic semialdehyde, urine purine and pyrimidines, copper, ceruloplasmin, CSF neurotransmitters and amino acids, urine for muchopolysaccharides, DNA for mitochondrial panel, CGH array and Infantile Epilepsy gene panel). Plasma amino acids showed increased s-sulfocysteine and alpha-amino adipic semialdehyde, with the rest of the amino acids essentially normal, suggestive of ISOD rather than molybdenum cofactor deficiency. The Infantile Epilepsy gene panel and parental genetics confirmed the diagnosis of autosomal recessive SOD with two pathogenic gene variants identified (NM_000456.2(SUOX):c.302G>A p.(Trp101Ter) and NM_000456.2(SUOX):c.1084G>A p.(Gly362Ser)). Confirmatory genetic results were available at approximately 25 days of life.

Developmentally at four and half months, she was fixing, following and cooing but not smiling. On prone positioning, she lifted her head briefly but could not maintain this position, with her elbows positioned posterior to her shoulders. She didn’t reach or bring her hands to the midline. Expected developmental milestones at four months include: spontaneous smiling, babbling, mimicking sounds and facial expressions, fixing and following, reaching for toys, holding head steady and unsupported, bringing hands to mouth, pushing up on elbows when prone and possible initiation of rolling over from tummy to back. At 6 months of age, the infant continued to experience seizures despite multiple antiseizure medications. Seizures were varied, occurring in clusters throughout the day, usually lasting between two to ten minutes. Some were characterised by dusky episodes associated with apnoea, while others were characterised by tonic bilateral limb stiffening, with intermittent myoclonic jerks and occasional eye involvement with flickering and deviation. The initial treatment was gradually changed and at four months of age, the infant was on a maintenance regime of Vigabatrin, Clonazepam and Sodium Valproate, with some reduction in seizures. She was entirely enteral fed and required hyoscine for management of ongoing secretions. On examination at 6 months of age, she had minimum spontaneous movements, significant truncal hypotonia with marked head lag on pulling to sit, with peripheral hypertonia and lower limb clonus bilaterally. She was microcephalic with a closed anterior fontanelle and prominent sutures. Unfortunately, due to clinical instability, the ophthalmologic exam could not be performed within 6 months of life.

## Consent

Written informed consent for publication of the clinical information was obtained from the child’s legal guardian.

## Literature review

On 4 ^th^ January 2021, we performed a literature search on PubMed for published cases with ISOD, using “sulfite oxidase deficiency” as a search term. The search was limited to publications in English language and resulted in a total of 106 papers. After title and abstract review, 41 papers were included for full review (one paper added from reference review). The same search was performed independently by another author (CMS) on 15 May 2021. Sixteen papers reporting a total of 19 newborns diagnosed with early onset ISOD which had any aEEG/EEG description were included in the final review (papers excluded: n=11 EEG pattern not described, n=9 presented late onset ISOD, n=3 abstract in English but not the full text, n=1 review paper, n=1 paper only abstract available). Gender, family history, age of onset and clinical presentation, seizure presence and management, EEG description, brain imaging details and outcome of all cases published are summarised in Table 1.

**Table 1.** Previous publishes cases of early onset isolated sulfite oxidase deficiency with electroencephalographic description (n=19 cases).

Author and publication year	No cases	Sex	Family history	Age of Onset (days)	Clinical presentation	Clinical seizures	Seizure management	Ophthalmological examination	EEG description	Brain imaging	Outcome
Duran *et al.* ^7^ 1981	1	M	Non-consanguineous parents	1	Onset: respiratory distress and seizures.	Twitching of facial muscles	Refractory to phenobarbital	No details	No electrical activity	CT unremarkable	Died at 8 days of life
Rupar *et al.* ^8^ 1996	1	M	Non-consanguineous parents	1	Onset: poor feeding, lethargy, central hypotonia and peripheral hypertonia, with seizures.	Tremulousness and bicycling movements	Refractory to treatment with phenobarbitone, valproic acid, phenytoin, pyridoxine and diazepam	Spherophakia	Diffuse abnormality with slowing but no localized epileptogenic focus	CT scan: ventriculomegaly, reduced gyri and widened sulci	Microcephaly. Severe developmental delay. Died at 32 months
Edwards *et al.* ^9^ 1999	2, brothers	M	Non-consanguineous parents	1	Onset: respiratory distress and seizures; tracheomalacia with poor feeding; hypertonicity, opisthotonos, fist clenching.	Myoclonus and repetitive cycling movements	Controlled with phenobarbital	Bilateral nasal subluxation of the lenses, exotropic eyes Not fixing and following. Sluggish pupillary reactions with no afferent defect, optic disc pallor poor foveal and nerve fiber layer reflexes	Electrographic seizures; no details of the background activity	MRI: Diffuse white matter abnormalities, extensive macrocystic changes; small basal ganglia; calcification in the cerebral peduncles; brainstem and cerebellum appeared hypoplastic with significant surrounding cisternal fluid.	No details
		M	Non-consanguineous parents	1	Onset: poor feeding, respiratory distress and refractory seizures with opisthotonos.	Yes, no description	Refractory to treatment (drugs not specified)	No details	Diffuse encephalopathy; seizures.	CT: white matter changes similar as the brother	Died at 10 months
Dublin *et al.* ^10^ 2002	1	F	Not stated	1	Onset: generalised seizures	Yes, no description	Controlled with phenobarbital	Normal	Diffuse, bilateral, hemispheric epileptiform discharges	MRI day 5: possible ischaemic pattern; MRI day 12: signal intensity abnormalities in the midbrain, thalamic, and basal ganglia, and diffuse white matter changes; MRI day 31: large cysts within the periventricular white matter, as well as cortical, brainstem, thalamic, and basal ganglia signal intensity abnormalities	At 3 months: severe developmental delay.
Hobson *et al.* ^11^ 2005	1	F	Non-consanguineous parents, older brother died from ISOD on day of life 20 but no EEG details available, one other healthy unaffected brother	1	Jerking movements 40 minutes following delivery	Generalised	Seizure activity and screaming episodes that were improved with the use of triclofos sodium	Normal	Day 28: discontinuous trace with inter- hemispheric asynchrony and prolonged periods of absent cortical activity.	Cranial ultrasound day 1: immature gyral pattern with no other significant abnormalities. CT day 2: extensive low attenuation in the white matter of both hemispheres, more than would be expected due to non-myelination. The corpus callosum was abnormal. MRI day 5: normal MRI day 18: Dramatic changes with cystic encephalomalacia involving the frontal, parietal and, to a lesser extent, temporal lobes MRI at 3 months demonstrated the ongoing progressive nature of the neurodegeneration. These changes were evident on serial cranial ultrasound.	At 4-months, microcephalic and hypertonic,subtle dysmorphic features, including sunken eyes and a prominent forehead, At 8 months, responded to tactile stimuli and appeared to have areas of hyperaesthesia. Died at 16 months.
Seidahmed *et al.* ^12^ 2005	1	M	Consanguineous parents (first cousins); first son died at 1 month of unknown causes; two maternal cousins with ISOD	2	Onset: refractory generalized seizure.	Yes, no description	Controlled with multiple antiseizure drugs (phenobarbital, phenytoin, midazolam)	Cortical blindness without lens dislocation	Diffuse bilateral hemispheric epileptiform discharges and no hypsarrhythmia	CT day 4: extensive low attenuation changes of the brain parenchyma, compatible with severe hypoxia. CT day 14: symmetrical widespread destructive lesions of the white matter of both hemispheres with cystic lesions. MRI 7 months: marked brain atrophy with signal intensity abnormalities in the mid-brain, thalamus and basal ganglia and large cysts within the periventricular white matter	At 6 months: dysmorphic features (narrow bifrontal diameter, deep- seated eyes and large ears); At 8 months: microcephaly, global neurodevelopmental delay, lack of visual fixation, truncal hypotonia with spasticity of all limbs, brisk reflexes and clonus.
Tan *et al.*/Eichler *et al.* ^13, 14^ 2005	1	M	Non-consanguineous parents; 3 maternal uncles and 2 paternal uncles died in infancy of unknown cause.	2	Onset: respiratory distress, poor feeding, hypotonia, opisthotonos, high-pitched cry; Day 4 apnoea associated with desaturation, seizure; generalized hypotonia with symmetric deep tendon reflexes and the presence of the Babinski reflex; then became hypertonic and hyperreflexic.	Bicycling movements in right foot and rhythmic tonic-clonic activity of right upper extremity	No response to phenobarbital, and phenytoin was added. He did not respond to a pyridoxine trial	Initially normal. At 11 months of age, bilateral mild nasal subluxation of the lenses.	Initial EEG: Diffuse, bilateral, hemispheric epileptiform discharges, predominantly over the frontotemporal regions, right greater than left. The background showed a marked burst suppression pattern. EEG at 2 weeks of life showed no organized seizure activity, but the burst suppression pattern continued. EEG at 3 months of age showed predominantly left-sided spike and sharp wave complexes, but no electrographic seizures.	CT day 4: loss of grey-white matter differentiation in the frontal, parietal and occipital regions bilaterally. The right caudate head and putamen were hypodense. MRI day 5: widespread decreased diffusion throughout the entire cortex, subcortical white matter and basal ganglia. MRI at 3.5 months: cystic changes in the cortical and subcortical white matter.	Microcephaly and severe developmental delayed. At 13 months: very hypertonic in all 4 limbs, with brisk deep tendon reflexes, but his head control and truncal tone were very poor; occasional opisthotonos and repeated startle responses to loud noises. Developmentally: unable to roll over or sit up, not reaching out for objects, with no meaningful vocalization.
Sass *et al.* ^15^ 2010	1	F	Non-consanguineous parents; sibling with similar clinical picture and brain MRI died at 21 months	3	Onset: poor feeding, hypoactivity, dyspnoea; status epilepticus three times a year; Neurological examination at 1 month: mild hypotonia, normal rooting, stepping and placing reflexes, which disappeared in second month.	Subtle and erratic clonic seizures; Status epilepticus.	Poorly controlled with phenobarbital, topiramate, carbamazepine	Normal at 2 years	Disorganized background activity and medium amplitude multifocal sharp waves, mainly in the Rolandic and frontal bilateral distribution, without hypsarrhythmia	CT: diffuse oedema; MRI day 37: multicystic leukoencephalomalacy and cerebellar atrophy	At 2 years: microcephaly, complete cervical and trunk hypotonia with rigid limbs (without knife or cogwheel signs), reduced general movements, no eye contact, no social smile, no signs of irritability. Epilepsy on topiramate, phenobarbital and carbamazepine, seizure still not controlled.
Holder *et al.* ^16^ 2014	1	M	Consanguineous parents (second cousins); sibling with ISOD;	1	Onset: seizures, marked, diffuse hypotonia;	Myoclonic jerks, tonic extension of all extremities	Poorly controlled with multiple antiseizure drugs (drugs not specified)	Microspherophakia	Initial EEG: low-amplitude background with multifocal electrographic seizures of multiple abnormalities; EEG 15 months: hypsarrhythmia; Further EEGs: continued multifocal sharp waves, short episodes of stiffening extremities with tactile stimulation (hyperekplexia) with no electrographic correlate, reduced in frequency with clonazepam; EEG at 15 months: extremely high voltage and chaotic background consistent with hypsarrhythmia, several brief tonic seizures with attenuation of hypsarrhythmia.	MRI on day 5 - widespread decreased diffusivity in the posterior frontal, parietal, occipital lobes bilaterally; MRS: abnormal elevation of lactate peaks in the bilateral basal ganglia, thalami, and occipital lobes; CT at 5 months: progressive degeneration of the brain with volume loss, cavitary lesions in the cerebral white matter and bi-thalamic micromineralization.	At 6 months: microcephaly, hypotonia with hyperreflexia, motor and speech delay At 15 months: infantile spasms.
Westerlinck *et al.* ^17^ 2014	1	F	Consanguineous parents (distant cousins);	2	Onset: intermittent episodes of hypertonia and hypotonia.	No seizure activity noted	-	No details	On day 2: no focal epileptic activity during repeated short contractions in both arms and/ or limbs. Repeat EEG day 9: diffusely slowed monomorphic trace of rather low voltage, but again without epileptic characteristics.	MRI day 3: hypoplasia of the corpus callosum, diffuse cystic degeneration of the supratentorial white matter, mainly involving the frontoparietal regions.	No details
Relinque *et al.* ^18^ 2015	1	M	Non-consanguineous parents	3	Onset: generalized hypertonia, poor reactivity, weak cry and poor suck, seizures. At 49 days: flexed limb posture, weak cry, thumb in fist posture, axial hypotonia, incomplete moro and poor suck;	Generalized tonic seizures: axial hypertonia and boxing movements in the legs; Status epilepticus.	Poorly controlled (drugs not specified)	No details	Initial aEEG status epilepticus, which required a drug-induced coma to control seizures. EEG: it was observed burst-suppression pattern;	MRI: diffuse hyperintensity in basal ganglia and cystic formations in white matter, thinned cerebral cortex; restricted diffusion in basal ganglia and corticomedullary junctions	Died at 2 months
Zaki *et al.* ^3^ 2016	3	M	Consanguineous parents	50	Onset: poor feeding and growth, intractable seizures, axial hypotonia and spastic quadriparesis; Hyperekplexia; facial dysmorphism (frontal bossing, depressed nasal bridge, anteverted nares, retrognathia, puffy checks and low-set ears).	Generalized tonic clonic and multifocal myoclonic	Refractory to multiple antiseizure treatment (drugs not specified)	Normal, no lens subluxation	Focal abnormalities	MRI: calcifications in the thalami; Subcortical cysts; Abnormal basal ganglia; Cerebral atrophy; wide interhemispheric fissure; thin corpus callosum; white matter loss; cerebellar atrophy, cystic encephalomalacia	Severe developmental delay; died before 2.5 years
		M	Consanguineous parents	15	Onset: poor feeding and growth, intractable seizures, axial hypotonia and spastic quadriparesis; Hyperekplexia; facial dysmorphism (frontal bossing, depressed nasal bridge, anteverted nares, retrognathia, puffy checks and low-set ears).	Generalized tonic clonic and multifocal myoclonic	Refractory to treatment (drugs not specified)	Normal, no lens subluxation	Hypsarrhythmia	MRI: calcifications in the thalami; Subcortical cysts; Cerebral atrophy; wide interhemispheric fissure; thin corpus callosum; white matter loss; cerebellar and brain stem atrophy, cystic encephalomalacia.	Severe developmental delay; died before 2.5 years
		F	Consanguineous parents; affected sibling	40	Onset: poor feeding and growth, intractable seizures, axial hypotonia and spastic quadriparesis;	Generalized tonic clonic and multifocal myoclonic	Refractory to treatment (drugs not specified)	Normal, no lens subluxation	Focal abnormalities	MRI: Subcortical cysts; Cerebral atrophy; thin corpus callosum; white matter loss; cerebellar and brain stem atrophy, cystic encephalomalacia.	Severe developmental delay
Lee *et al.* ^19^ 2017	1	F	Non-consanguineous parents	1	Onset with poor feeding; day 5 decreased activity; day 8: subtle seizures, high- pitched crying; neurological examination poor eye contact, intact cranial nerves except for poor sucking and swallowing power, brisk deep tendon reflexes with extensor plantar reflex, a positive ankle clonus, generalized hypertonicity, rigidity, intermittent dystonic posture.	Bicycling of legs, alternating myoclonic seizures with rhythmic jerking over limbs	Refractory to multiple antiseizure treatment (drugs not specified)	Normal at 2 years and 3 months	Diffuse low amplitude background activity.	MRI day 9: ventricular dilatation, cystic lesions over the left frontal and temporal areas, diffuse and evident T2 high signal intensity of the bilateral cerebral cortex, and increased T2 signal intensity of the globus pallidi; MRS: inverted lactate peak; MRI at 4 months: cerebral cortical atrophy, multiple and small cystic lesions over bilateral occipital areas, subdural haemorrhage over the left frontal and temporal areas.	2 years 4 months old and bedbound with rigid limbs, intermittent, evident dystonic posture along with screaming episodes, and no eye contact, refractory myoclonic seizures and multifocal seizures, dysmorphic face with microcephaly.
Bender *et al.* ^20^ 2019	1	M	Consanguineous parents	3	Onset with hypopnea and multiple seizures, comatose, requiring invasive ventilation for 4 days. After extubation remained neurologically abnormal, with hyperexitability, dyskinetic movements, spasticity, epileptic seizures and frequent vomiting.	Yes, no description	Refractory to vitamin B6, phenobarbitone, levetiracetam and sultiame, but responsive to topiramate.	No details	Reduced activity and multifocal epileptic discharges as well as focal epileptic activity.	MRI globally severely impaired diffusion, white matter hyperintensity in T2, cerebella hypoplasia; MRI day 7: diffuse supratentorial leukoencephalopathy with impaired diffusion and progressive brain oedema; MRI at 3 months: severe brain atrophy with multicystic leukoencephalopathy, epidural and subdural hygromas and large infratentorial CSF spaces; MRI at 5 months: worsened atrophy, microcephaly and hygromas; brainstem was relatively spared and showed signs of myelination.	At 4.5 years: microcephaly, severe developmental delay, epilepsy with seizures resembling myoclonic fits and dyskinesia.
Boyer *et al.* ^21^ 2019	1	F	Non-consanguineous parents	7	Onset: poor feeding, irritability; hypertonicity, opisthotonos and status epilepticus.	Yes, no description. Status epilepticus.	Partially controlled with phenobarbital, topiramate, and pyridoxine	Bilateral intraretinal haemorrhages; Exam at 3 months no lens subluxation.	Confirmed electroclinical and subclinical seizures; no details on the background activity.	MRI day 8: diffusely abnormal signal in the periventricular white matter, of both cerebral hemispheres, increased signal on the FLAIR sequences and the diffusion-weighted sequence in both temporal and parietal cortex and thinning of the corpus callosum; MRI week 6: severe global volume loss, cystic encephalomalacia, and bilateral moderate subdural effusions.	Severe developmental delay and died at 4 months of age.
Scramstad *et al.* ^22^ 2020	1	M	Not stated	3	Onset: intractable seizures	Yes, no description	Refractory to antiseizure treatment (drugs not specified)	No details	Frequent multifocal electrographic and electroclinical seizures.	MRI brain: diffuse diffusion restriction mimicking hypoxic ischaemic injury	Not stated

All infants were born at term after an unremarkable pregnancy and delivery. Out of the 19 infant cases, the majority were males (12 infants) and family history of consanguinity and/or similar medical history in other family members was present in nine infants. Except for a cohort of three infants published by Zaki *et al*. ^3^, in all infants the onset was within the first week of life. The initial clinical presentation was characterised by a combination of respiratory distress, hypotonia with poor feeding and seizures. Except for one case, all infants presented with early seizures, the majority were refractory to multiple antiseizure drugs and seizures and even status epilepticus persisted in most infants despite treatment. In 18 infants, clinical seizures were noted, however seizure semiology was described in only 13 infants, and ‘tonic-clonic’ and ‘myoclonic’ seizures were most commonly described. In addition, many described bicycling movements of the lower limbs. In two infants subtle seizures were also described. Ophthalmologic abnormalities, especially lens subluxation, are frequently present later in infancy in ISOD and might be an important diagnostic clue mainly for late onset SOD ^4^. In the cohort described in this review, 11 infants had eye examination described and 5 have some abnormalities but only one infant had lens subluxation. This could be explained by the severity of the cases and early death.

All 19 infants reported and included in this review had some aEEG or EEG monitoring performed, however details regarding the monitoring are lacking (start and duration of monitoring, recording electrodes used). Although clinical seizures were noted in the majority of infants, electrographic seizures were confirmed in only 10 infants and described as frequent, multifocal epileptic discharges. However, electrographic seizures could have been missed if the EEG monitoring was performed intermittently for short periods of time (continuous EEG monitoring is rare for infants without hypoxic ischaemic encephalopathy). Status epilepticus was reported in only 3 infants. EEG background patterns were described in 14 cases and varied from diffuse encephalopathic patterns (from low amplitude background to burst suppression), to a pattern of hypsarrhythmia and even isoelectric tracings.

All infants had brain imaging performed and, except for one infant that died early, multiple brain abnormalities were reported, including diffuse white matter changes, calcifications, evolving rapidly to cystic lesions and atrophy.

The prevalence of ISOD is unknown (approximately 50 cases described in the literature), but it is known to have a devastating prognosis, especially for the early onset (classical) ISOD. Three infants in the 19 included in our review had no follow up reported, however all the remaining infants had severe developmental delay and seven died before 3 years of age. 

## Discussion

To our knowledge, this is the first case report of a newborn diagnosed with early onset ISOD where prolonged continuous EEG monitoring in the neonatal period was also recorded and described. Two recently published literature reviews have summarised different aspects of newborns diagnosed with ISOD, but our main focus was to describe and summarise the evolution of neonatal electroencephalographic patterns in ISOD ^5, 6^.

Similar with the majority of cases presented in the literature, our infant was born in good condition at term after an uneventful pregnancy, with early signs of encephalopathy, seizure activity and with an initial brain MRI showing severe and diffuse abnormalities, mimicking hypoxic-ischaemic injury. The majority of cases presented in the literature had clinical seizures and in approximately half of the infants (10 out of 19 infants), the seizures were confirmed on EEG, with an EEG background activity which varied from low amplitude background to burst suppression, to hypsarrhythmia pattern and even isoelectric pattern. In the case presented here, the initial EEG background pattern showed a diffuse encephalopathic pattern with no sleep cycling and frequent multifocal seizures. The evolution of the EEG background activity has deteriorated over the first week of life, which may be explained by a combination between the evolution of the disease and the escalation of anti-seizure treatment. The hourly seizure activity ( Figure 3) showed a decrease in seizure burden after the first 72 hours likely due to anti-seizure treatment. After close evaluation of the background pattern described in our patient a pattern of slow waves with superimposed fast rhythmic activity (delta-beta complexes) was quite distinctive ( Figure 4). As stated these may represent a waveform more alike to the ‘Delta Crown’ waveform described by Flitton *et al.* ^2^, in Molydenum cofactor deficiency (MoCD). If the same, this raises the possibility that these waveforms are also a diagnostic marker in ISOD. It would be of interest to determine if this waveform is described in future cases of ISOD.

In some cases presented in the literature, including in our infant, the early MRI picture was similar to the picture found in severe hypoxic-ischaemic encephalopathy. However, in a newborn with seizures refractory to treatment and hypoxic-ischaemic injury on early MRI but without a clear perinatal hypoxic event, a diagnosis of ISOD could be considered ^10^. It has been shown with brain MRI studies that the evolution of the disease is towards progressive brain destruction, with cystic changes and atrophy.

Classic ISOD and MoCD are both autosomal recessive inborn errors of the metabolism of sulphated amino acid: an isolated defect of sulfite oxidase enzyme as in ISOD or in combination with defect of xanthine dehydrogenase enzyme as in MoCD. Both conditions have a similar clinical phenotype and a poor long term prognosis ^3^. In our case, the diagnostic genetic results were available only after three weeks of life, which warrants the need for rapid genetic testing in a case of intractable seizure in a newborn with encephalopathy. 

In conclusion, the infant presented in our report had early onset ISOD, with intractable seizures and evolving encephalopathy, a severely encephalopathic EEG pattern with distinct delta-beta complexes and frequent multifocal epileptic discharges and abnormal MRI. Previously reported cases in the literature described a similar EEG pattern with an encephalopathic background and refractory seizures. However, we presented in detail the early evolution of EEG background in ISOD with a possible diagnostic marker represented by the described delta-beta complexes. 
