# Supplementary material for: Case Report: Electroencephalography in a neonate with isolated sulfite oxidase deficiency : – a case report and literature review
Source: HRB Open Res. 2021 Nov 23;4:122. [Version 1] doi: 10.12688/hrbopenres.13442.1 (PMC8669778; doi:10.12688/hrbopenres.13442.1)
Supplement: Peer review history

# Peer review: Reviewer response for version 1

**DOI:** 10.21956/hrbopenres.14650.r30896

**Published:** 2021-12-13

**Authors:** Courtney Wusthoff

**Affiliations:** 1 Departments of Neurology and Pediatrics, Stanford University, Stanford, CA, USA

This report presents a case of isolated sulfite oxidase deficiency (ISOD) in a neonate. The authors focus on the evolution of EEG findings, captured through continuous EEG monitoring over a week period of a term neonate with ISOD. Also described is the clinical presentation, early MRI findings, and follow up to 6 months of age.

 A strength of this report is the thorough review of literature to date reporting findings in this condition, with excellent context provided both in the introduction and in the discussion. The figures are high quality, and clearly illustrate the points discussed.

 The authors propose that specific delta-beta complexes on EEG might be a diagnostic marker in this condition. These are shown from the case reported, and the authors refer to similar findings reported in cases of MoCD. They correctly point out it would be helpful in future cases reported of ISOD to identify whether this finding is always present on EEG. Particularly given the typical delay in confirming a genetic diagnosis, if this were to be replicated it could be a useful early clue as to etiology for newborns with refractory seizures.

 As such, it would also be important to consider whether this pattern has been reported in any other conditions - the authors briefly mention mechanical delta brushes as seen in preterm neonates, but do not state whether a literature review was performed to search for other causes of the pattern. My only suggestion to build upon this excellent report would be to include that detail.

Are enough details provided of any physical examination and diagnostic tests, treatment given and outcomes?

Yes

Is the case presented with sufficient detail to be useful for other practitioners?

Yes

Is sufficient discussion included of the importance of the findings and their relevance to future understanding of disease processes, diagnosis or treatment?

Yes

Is the background of the case’s history and progression described in sufficient detail?

Yes

Reviewer Expertise:

Neonatal neurology, EEG

I confirm that I have read this submission and believe that I have an appropriate level of expertise to confirm that it is of an acceptable scientific standard.

# Peer review: Reviewer response for version 1

**DOI:** 10.21956/hrbopenres.14650.r30897

**Published:** 2021-12-02

**Authors:** Fabrice Wallois

**Affiliations:** 1 INSERM UMR-S 1105, GRAMFC, Université de Picardie-Jules Verne, Amiens, France

This is a case report of EEG in a neonate with isolated sulfite oxidase deficiency. This is a very well documented case report which is nicely presented. This is a rare pathology that very interestingly shares with the MoCD (another autosomal recessive inborn errors of the metabolism of sulphated amino acid) the delta-beta complexes and frequent multifocal epileptic discharges with an abnormal nonspecific MRI. I do agree that these delta-beta complexes, which have only been described once and which I have never seen, might be considered as a very specific neurobiomarker of ISOD and/or MoCD. 

 I have only very few comments on this interesting well done case report:

- Labelling of the EEG channels in the different figures is hard to read. An alternative would be to indicate the order of the channels in the legend. I suppose that the upper traces are from aEEG. Because they are not analyzed, I propose to suppress them as they do not provide additional information’s’. Or you should extract the meaningful information.
- In the case presentation, the evolution is not clear for me. The paragraph starting with “Developmentally at four and half month…” is not chronologically organized. For example at 4.5 month the neonate does not smile and she did not brings her hands to the midline. But I do not understand the objective of the paragraph starting with “Expected developmental…..” in which information’s seems contradictory. Then at 6 month she still have seizure but this was not indicated at 4 and 4.5 months. I would be very interested to know the follow up for this child. If some information’s can be add at the end of the review process that would be nice. 

I have no other comments on this interesting case report.

Are enough details provided of any physical examination and diagnostic tests, treatment given and outcomes?

Yes

Is the case presented with sufficient detail to be useful for other practitioners?

Yes

Is sufficient discussion included of the importance of the findings and their relevance to future understanding of disease processes, diagnosis or treatment?

Yes

Is the background of the case’s history and progression described in sufficient detail?

Yes

Reviewer Expertise:

Neonatal clinical neurophysiology

I confirm that I have read this submission and believe that I have an appropriate level of expertise to confirm that it is of an acceptable scientific standard.
